# Strategies for discontinuing vasopressin and norepinephrine during the recovery phase of shock: a single-center retrospective study

**DOI:** 10.1186/s40560-025-00823-w

**Published:** 2025-09-30

**Authors:** Shiho Suganuma, Shigehiko Uchino, Seiya Nishiyama, Yusuke Sasabuchi, Shinshu Katayama

**Affiliations:** 1 Department of Emergency Medicine, Yamagata Prefectural Central Hospital, 1800, Aoyagi, Yamagata, Yamagata 990-2292 Japan; 2 Department of Anesthesiology and Critical Care Medicine, Jichi Medical University Saitama Medical Center, 1-847, Amanuma-Cho, Omiya-Ku, Saitama, Saitama 330-0834 Japan; 3 Department of Real-World Evidence, Graduate School of Medicine, The University of Tokyo, 7-3-1, Hongo, Bunkyo-Ku, Tokyo, 113-0033 Japan

**Keywords:** Arginine vasopressin, Hypotension, Norepinephrine, Overlap weighting, Shock, Tapering, Vasopressor discontinuation

## Abstract

**Background:**

The optimal strategy for discontinuing arginine vasopressin and norepinephrine in patients recovering from shock remains uncertain. Although prior studies have suggested a higher risk of hypotension when arginine vasopressin is discontinued first, these findings may have been influenced by baseline imbalances and tapering practices. We conducted a retrospective study to evaluate whether the order of discontinuation between arginine vasopressin and norepinephrine was associated with the incidence of hypotension during the recovery phase of shock, with vasopressor end doses converted to norepinephrine equivalents for analysis.

**Methods:**

This was a single-center retrospective cohort study of intensive care unit patients with shock who received both arginine vasopressin and norepinephrine from August 2017 to March 2024. Patients were categorized based on whether arginine vasopressin or norepinephrine was discontinued first. The primary outcome was the incidence of hypotension within 24 h of vasopressor cessation, defined as mean arterial pressure < 60 mmHg requiring a ≥ 25% increase in the remaining vasopressor, reinstitution of the stopped agent, or a bolus of ≥ 500 mL crystalloid or 25 g albumin. Overlap weighting using propensity scores was applied to adjust for baseline imbalances both in the overall cohort and in the septic shock subgroup. Propensity scores were estimated using logistic model, including baseline characteristics, hemodynamic parameters, and vasopressor end doses in norepinephrine equivalents.

**Results:**

A total of 524 patients were analyzed, with 293 discontinuing AVP first and 231 discontinuing NE first. In the unadjusted cohorts, hypotension occurred in 19% of the AVP-first group and 26% of the NE-first group. After overlap weighting, all baseline covariates were balanced between the groups, and the incidence of hypotension was not significantly different (19% vs 21%, *P* = 0.59). In the septic shock subgroup (*n* = 267), the weighted analysis showed no significant difference in the incidence of hypotension between groups.

**Conclusions:**

In patients recovering from shock who received both arginine vasopressin and norepinephrine, discontinuing arginine vasopressin first was not associated with a higher risk of hypotension.

**Supplementary Information:**

The online version contains supplementary material available at 10.1186/s40560-025-00823-w.

## Background

Shock, a state of circulatory failure resulting in inadequate cellular oxygen utilization [1], is a common condition in the intensive care unit (ICU) [2]. Vasopressors are frequently administered to manage shock, especially vasodilatory shock such as septic shock, post-cardiovascular surgery, post-acute myocardial infarction, and shock following general surgery and anesthesia [3, 4]. The Surviving Sepsis Campaign Guidelines 2021 recommend norepinephrine (NE) as the first-line vasopressor for septic shock; for patients with inadequate mean arterial pressure (MAP) < 65 mmHg, arginine vasopressin (AVP) should be added rather than increasing the NE dose [5].

Despite established guidelines for initiating vasopressor therapy, optimal discontinuation strategies remain unresolved. Several studies reported that discontinuing AVP before NE may be associated with a higher incidence of hypotension [6–10]. However, these findings are limited by potential confounding due to baseline imbalances and differences in tapering practices. NE is generally tapered gradually, whereas AVP is often discontinued abruptly [6–10], possibly influencing outcomes.

To address these limitations, we conducted a retrospective observational study applying overlap weighting with propensity scores to minimize confounding [11]. In addition, we converted the AVP end dose into NE equivalents to compare vasopressor end doses on a consistent scale. The aim of this study was to evaluate whether the order of discontinuation between AVP and NE is associated with the incidence of hypotension during the recovery phase of shock.

## Methods

### Study design and setting

This single-center retrospective cohort study was conducted in the ICU of Jichi Medical University Saitama Medical Center to investigate the association between the discontinuation order of AVP and NE with the occurrence of hypotension during the recovery phase of shock. The study was approved by the Institutional Review Board of the Jichi Medical University Saitama Medical Center (No. S24-144) and conducted in accordance with the amended Declaration of Helsinki. Informed consent was obtained through an opt-out process.

### Participants

We screened all ICU admissions from August 1, 2017, to March 31, 2024. Inclusion criteria were: age ≥ 18 years, diagnosis of shock, initiation of both AVP and NE within 24 h of admission, and concurrent use of both agents. Exclusion criteria were: simultaneous discontinuation of AVP and NE, absence of discontinuation of either AVP or NE, or use of additional vasopressors at the time of complete discontinuation (cessation). Patients were classified into two mutually exclusive groups, those in whom AVP was discontinued first and those in whom NE was discontinued first, based on clinician discretion.

### Vasopressor management in clinical practice

Vasopressor tapering or cessation was primarily guided by mean arterial pressure at the discretion of the treating physicians. Norepinephrine was typically titrated by ICU nurses per protocol, with the dose usually reduced by 0.1 mL/h when MAP > 70 mmHg, corresponding to approximately 0.03–0.12 mg/h depending on the drug concentration. Arginine vasopressin was generally managed by physicians, rather than titrated by nurses.

### Data collection and measurements

Data were retrospectively collected from the hospital’s electronic patient management system for critical care (ACSYS-Ki^™^, PHILIPS Japan, Tokyo, Japan). Baseline demographic and clinical data were collected at ICU admission, including ICU admission route, primary diagnosis, chronic comorbidities (respiratory failure, liver cirrhosis, hematological malignancy, cancer metastasis, immunosuppression, maintenance dialysis), Acute Physiology and Chronic Health Evaluation (APACHE) III score, serum creatinine level, serum lactate level, and PaO_2_/F_I_O_2_ ratio. At the time of cessation of the first vasopressor, we collected serum lactate level, Sequential Organ Failure Assessment (SOFA) score (cardiovascular components excluded), and the use of intra-aortic balloon pumping (IABP), veno-venous extracorporeal membrane oxygenation (VV-ECMO), veno-arterial extracorporeal membrane oxygenation (VA-ECMO), and renal replacement therapy (RRT). Hemodynamic parameters, including MAP, heart rate (HR), cumulative fluid balance, and atrial fibrillation occurrence were recorded both at first vasopressor cessation and within 24 h thereafter. Data of all pharmacological agents that could influence the occurrence of hypotension, including AVP and NE dosing, other vasopressors, and corticosteroids, were collected. Laboratory data at ICU admission and at first vasopressor cessation were obtained within 24 h before each respective timepoint. Hemodynamic and supportive treatment parameters were automatically recorded every hour through the electronic medical record system and reflect near real-time clinical status. For consistency in analysis, AVP doses were converted to NE equivalents using the following formula: AVP (U/min) × 2.5 = NE (μg/kg/min) [12]. In addition, we classified vasopressor discontinuation as either abrupt or gradual weaning. Abrupt discontinuation was defined as stopping the drug at its maximum dose, while gradual weaning was defined as discontinuation after dose reduction below the maximum, following definitions used in the prior study [13]. There were no missing data except for height (*n* = 47) and weight (*n* = 49), which were imputed using median values.

### Outcome measures

The primary outcome was the incidence of hypotension within 24 h after the first vasopressor cessation. Hypotension was defined as MAP under 60 mmHg with any of the following interventions: an increase of 25% or more in the remaining vasopressor dose, reinstitution of the discontinued agent, or administration of a fluid bolus of at least 500 mL of crystalloid or 25 g of albumin. Secondary outcomes included hospital mortality, ICU mortality, hospital length of stay, ICU length of stay, incidence of new-onset atrial fibrillation, cumulative fluid balance from the first vasopressor cessation to ICU discharge, and total vasoactive medication duration.

### Statistical analysis

As this was a retrospective database study, we included all eligible patients from the registry rather than performing a formal sample size calculation. Baseline characteristics and clinical outcomes were summarized for each group: categorical variables as count (percentages), and continuous variables as median (25–75% interquartile range). Wilcoxon’s rank sum test or χ-square test were used to compare between groups. To address confounding, we applied overlap weighting approach based on a propensity score estimated using a logistic regression model in which the treatment group was the dependent variable [11]. Covariates included age, sex, weight, height, primary diagnosis, chronic comorbidities, ICU admission type, corticosteroid use, APACHE III score, SOFA score (cardiovascular components excluded), laboratory values at admission (serum creatinine, lactate, PaO_2_/F_I_O_2_ ratio) and lactate at vasopressor cessation, cumulative fluid balance before cessation, use of RRT, hemodynamic parameters at cessation (MAP, HR, and use of IABP, VV-ECMO, VA-ECMO), vasopressor end dose (defined as the final dose of the discontinued agent—either AVP or NE—immediately before cessation), the remaining vasopressor dose at cessation, and the duration from ICU admission to first vasopressor cessation. Quadratic terms for continuous variables (weight, PaO_2_/F_I_O_2_ ratio at admission, lactate at cessation, vasopressor end dose in NE equivalent) and an interaction term between APACHE III score and SOFA score were included to improve model specification. Covariate balance was assessed by standardized mean difference (SMD), with SMD less than 0.1 indicating adequate balance. The distributions of propensity scores before and after weighting are shown in Figure S1. In weighted cohorts, we compared outcomes using weighted logistic or linear regression for dichotomous or continuous outcomes, respectively.

To assess the robustness of our findings, we performed a sensitivity analysis excluding patients receiving mechanical circulatory support (IABP, VV-ECMO, or VA-ECMO) at the time of vasopressor cessation. Two subgroup analyses were also conducted among patients with septic shock and cardiogenic shock. Septic shock was defined as an infectious primary diagnosis and a serum lactate level over 2 mmol/L. Cardiogenic shock was defined as cases with a primary diagnosis categorized under cardiovascular conditions. For the septic shock subgroup, the same propensity score model was applied and overlap weighting was performed as described above. Due to the limited sample size in the cardiogenic shock subgroup, we did not perform propensity score weighting and instead report unadjusted results.

In addition to the main analysis, we performed an exploratory comparison of abrupt vs tapered AVP discontinuation among patients in the AVP discontinued first group. Descriptive and unadjusted analyses were used to support interpretation of the main findings, without aiming to infer causality. All statistical analyses were performed using the R software (version 4.4.2; R Foundation for Statistical Computing, Vienna, Austria).

## Results

**Fig. 1:**
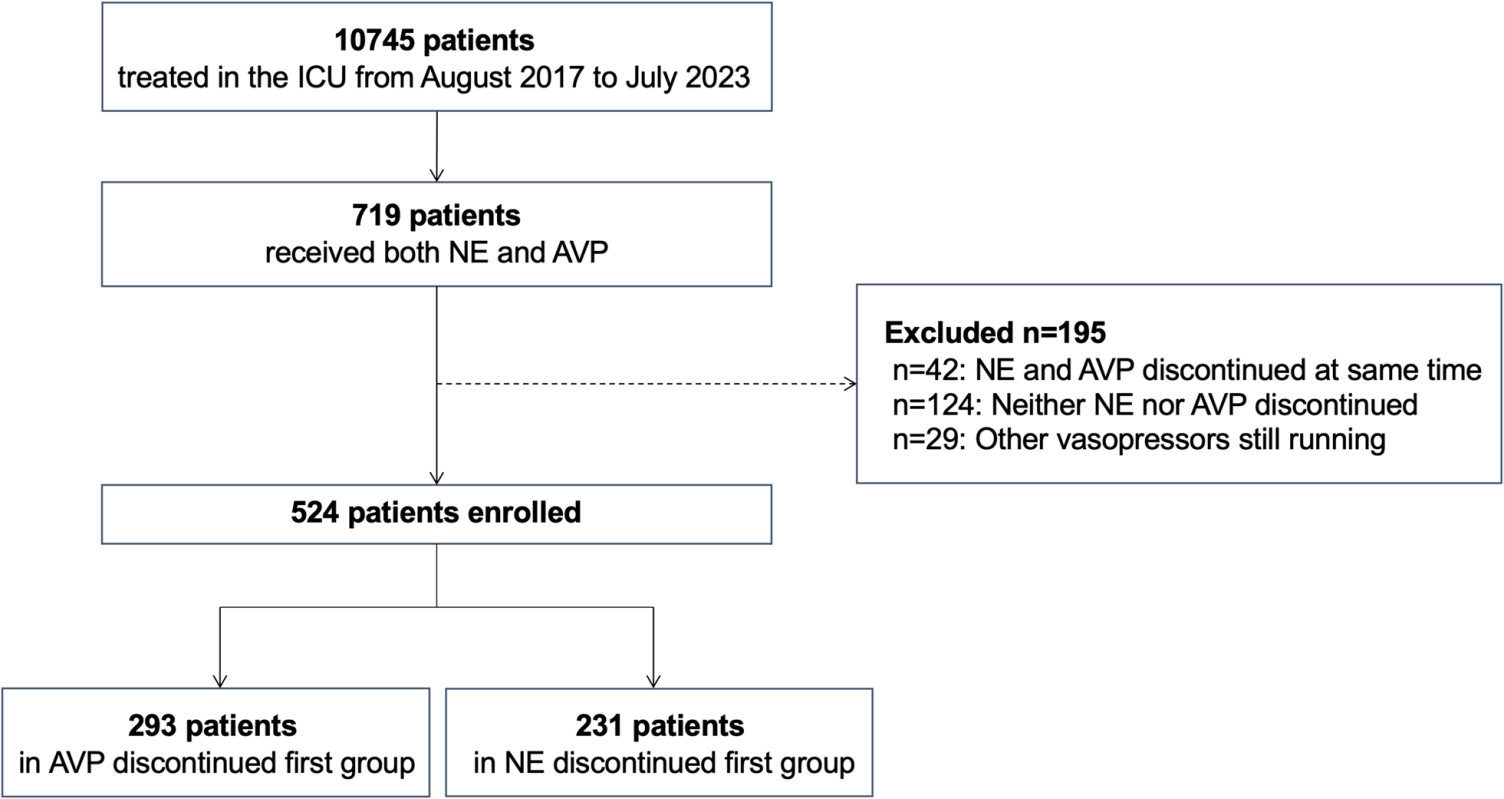
Patients flow. AVP: arginine vasopressin; NE: norepinephrine

A total of 10,745 patients were screened, and 719 patients met the inclusion criteria. After the exclusion criteria were applied, 524 patients were enrolled for analysis, with 293 discontinuing AVP first and 231 discontinuing NE first (Fig. 1). Baseline characteristics of unadjusted and weighted cohorts are shown in Table 1. Table 2 presents clinical characteristics, hemodynamic parameters, and vasopressor dosing at the time of the first vasopressor cessation, including the vasopressor end dose and the remaining vasopressor dose. In the AVP discontinued first group, 67% underwent gradual weaning of AVP; in the NE discontinued first group, 84% weaned. After overlap weighting, all variables were balanced between the two groups.

**Table 1:** Baseline characteristics at ICU admission (before and after overlap weighting)

Variables	Unadjusted cohort				Weighted cohort		
	Overall *n* = 524	AVP first *n* = 293	NE first *n* = 231	SMD	AVP first	NE first	SMD
Age (year)	71 (61–79)	72 (63–79)	70 (60–79)	0.089	72 (60–79)	71 (61–80)	< 0.001
Admission type							
Emergency Department	179 (34)	100 (34)	79 (34)	< 0.001	25.4 (33)	25.4 (33)	< 0.001
Elective surgery	32 (6)	19 (7)	13 (6)	0.009	5.3 (7)	5.3 (7)	< 0.001
Emergency surgery	74 (14)	41 (14)	33 (14)	0.003	10.9 (14)	10.9 (14)	< 0.001
Transferred	41 (8)	21 (7)	20 (9)	0.015	4.6 (6)	4.6 (6)	< 0.001
Ward	198 (38)	112 (38)	86 (37)	0.010	30.9 (40)	30.9 (40)	< 0.001
Male	364 (70)	210 (72)	154 (67)	0.050	53.6 (69)	53.6 (69)	< 0.001
Weight (kg)	58.5 (50.0–69.1)	58.5 (50.6–69.0)	59.6 (49.6–70.0)	0.050	58.5 (50.9–69.4)	59.4 (50.0–68.9)	< 0.001
Height (cm)	162 (157–170)	162 (157–170)	163 (155–170)	0.073	162 (157–170)	164 (155–170)	< 0.001
Disease category							
Cardiovascular	88 (17)	44 (15)	44 (19)	0.040	13.0 (17)	13.0 (17)	< 0.001
Respiratory	130 (25)	77 (26)	53 (23)	0.033	17.3 (22)	17.3 (22)	< 0.001
Gastrointestinal	129 (25)	65 (22)	64 (28)	0.055	20.2 (26)	20.2 (26)	< 0.001
Neurological	17 (3)	8 (3)	9 (4)	0.012	2.5 (3)	2.5 (3)	< 0.001
Others	160 (31)	99 (34)	61 (26)	0.074	24.3 (32)	24.3 (32)	< 0.001
Chronic diseases							
Respiratory Failure	8 (2)	5 (2)	3 (1)	0.004	0.5 (1)	0.5 (1)	< 0.001
Liver Cirrhosis	15 (3)	2 (1)	13 (6)	0.050	1.2 (2)	1.2 (2)	< 0.001
Hematological Malignancy	45 (9)	28 (10)	17 (7)	0.022	6.2 (8)	6.2 (8)	< 0.001
Cancer Metastasis	20 (4)	16 (6)	4 (2)	0.037	1.8 (2)	1.8 (2)	< 0.001
Immunosuppression	83 (16)	47 (16)	36 (16)	0.005	12.0 (16)	12.0 (16)	< 0.001
Maintenance Dialysis	31 (6)	18 (6)	13 (6)	0.005	4.4 (6)	4.4 (6)	< 0.001
APACHE III score	105 (87–126)	106 (88–126)	104 (87–125)	0.008	104 (88–125)	104 (83–128)	< 0.001
Creatinine (mg/dL)	0.9 (0.9–1.8)	0.9 (0.9–1.8)	0.9 (0.9–1.7)	0.063	1.0 (0.9–1.9)	1.0 (0.9–1.7)	< 0.001
Lactate (mmol/L)	1.9 (1.3–4.1)	1.9 (1.3–4.1)	1.7 (1.3–4.1)	0.041	2.0 (1.3–4.6)	1.8 (1.3–5.0)	< 0.001
PaO_2_/F_I_O_2_	265 (194–265)	265 (182–265)	265 (225–265)	0.104	265 (199–265)	265 (193–273)	< 0.001
Cortisol use	400 (76)	231 (79)	169 (73)	0.057	60.1 (78)	60.1 (78)	< 0.001

Categorical variables are presented as n (%), and continuous variables are presented as median (IQR)

APACHE: acute physiology and chronic health evaluation; AVP: arginine vasopressin; NE: norepinephrine; SMD: standardized mean difference

**Table 2:** Variables at the first vasopressor cessation (before and after overlap weighting)

Variables	Unadjusted cohort				Weighted cohort		
	Overall *n* = 524	AVP first *n* = 293	NE first *n* = 231	SMD	AVP first	NE first	SMD
Lactate (mmol/L)	2.0 (1.4–3.3)	1.9 (1.4–3.1)	2.0 (1.5–3.4)	0.105	1.9 (1.4–2.9)	1.9 (1.3–3.0)	< 0.001
SOFA Total without cardiovascular	8 (6–11)	8 (6–11)	8 (6–11)	0.009	8 (6–10)	8 (6–10)	< 0.001
SOFA Respiratory system	2 (1–3)	2 (1–3)	2 (1–2)	0.047	2 (1–2)	2 (1–2)	0.034
SOFA Coagulation	1 (0–2)	1 (0–2)	1 (0–2)	0.096	2 (0–2)	2 (0–2)	0.059
SOFA Liver	0 (0–1)	0 (0–1)	0 (0–1)	0.088	0 (0–1)	0 (0–1)	0.062
SOFA Central nervous system	3 (2–4)	3 (2–4)	3 (2–4)	0.022	2 (2–3)	2 (2–3)	0.045
SOFA Renal function	2 (0–4)	2 (0–3)	2 (0–4)	0.047	1 (0–3)	1 (0–3)	0.030
IABP used	6 (1)	4 (1)	2 (1)	0.005	0.5 (1)	0.5 (1)	< 0.001
VV-ECMO used	9 (2)	3 (1)	6 (3)	0.016	0.4 (1)	0.4 (1)	< 0.001
VA-ECMO used	16 (3)	12 (4	4 (2)	0.024	1.4 (2)	1.4 (2)	< 0.001
RRT used	119 (23)	71 (24)	48 (21)	0.035	17.0 (22)	17.0 (22)	
Mean Arterial Pressure (mmHg)	74 (68–81)	73 (68–80)	75 (69–81)	0.117	74 (69–82)	76 (71–81)	< 0.001
Heart Rate (bpm)	87 (75–102)	87 (75–100)	87 (74–103)	0.042	85 (72–96)	84 (71–100)	< 0.001
Cumulative fluid balance before cessation (mL)	3186 (1455–5777)	3424 (1611–6112)	2872 (1373–5543)	0.093	3222 (1558–5774)	3063 (1651–5580)	< 0.001
Vasopressor end dose in NEE (μg/kg/min)	0.04 (0.02–0.04)	0.04 (0.02–0.04)	0.03 (0.02–0.10)	3.389	0.02 (0.02–0.04)	0.03 (0.02–0.04)	< 0.001
The other vasopressor dose at cessation in NEE (μg/kg/min)	0.05 (0.04–0.09)	0.08 (0.04–0.15)	0.04 (0.04–0.06)	2.734	0.04 (0.02–0.07)	0.04 (0.04–0.06)	< 0.001
Duration from ICU admission to first vasopressor cessation (hours)	29 (14–54)	29 (16–50)	30 (12–58)	0.101	30 (16–50)	36 (18–57)	< 0.001

Categorical variables are presented as n (%), and continuous variables are presented as median (IQR)

SOFA: sequential organ failure assessment; IABP: intra-aortic balloon pumping; VV-ECMO: veno-venous extracorporeal membrane oxygenation; VA-ECMO: veno-arterial extracorporeal membrane oxygenation; RRT: renal replacement therapy; NEE: norepinephrine equivalent: AVP (U/min) × 2.5 = NE (μg/kg/min); ICU: intensive care unit; AVP: arginine vasopressin; NE: norepinephrine; SMD: standardized mean difference

The clinical outcomes of the unadjusted and weighted cohorts are summarized in Table 3. In the unadjusted cohorts, hypotension occurred in 19% of the AVP discontinued first group and 26% of the NE discontinued first group. After weighting, the incidence of hypotension was 19% vs 21% (odds ratio 1.2, 95%CI 0.7, 2.0; risk difference 2.5%, 95%CI − 6.5, 11.5; *P* = 0.59). Secondary outcomes also showed no significant differences between the groups in weighted cohorts.

**Table 3:** Summary of clinical outcomes

	Unadjusted cohort		Weighted cohort			
	AVP first	NE first	AVP first	NE first	Effect estimates (95%CI)	*P*
Primary outcome						
Hypotension	19	26	19	21	OR 1.2 (0.7, 2.0); RD 2.5 (− 6.5, 11.5)	0.59
Secondary outcome						
Hospital mortality	40	48	34	40	OR 1.3 (0.8, 2.0); RD 5.7 (− 4.9, 16.4)	0.29
ICU mortality	26	30	19	23	OR 1.3 (0.8, 2.2); RD 4.5 (− 4.5, 13.4)	0.33
Hospital length of stay (days)	54	43	52	47	MD − 4.9 (− 20.2, 10.5)	0.53
ICU length of stay (days)	14	13	14	12	MD − 1.5 (− 5.1, 2.1)	0.41
Incidence of new-onset atrial fibrillation	12	12	9	14	OR 1.5 (0.7, 3.1); RD 4.1 (− 3.1, 11.3)	0.27
Cumulative fluid balance after cessation (mL)	4790	2886	3675	2262	MD − 1412.6 (− 3606.6, 781.3)	0.21
Total vasoactive medication duration (days)	7	5	6	5	MD − 0.7 (− 3.2, 1.8)	0.57

Categorical variables are presented as percentages, and continuous variables as means. Effect estimates are presented as odds ratios (ORs) and risk differences (RDs) for binary outcomes, and as mean differences (MDs) for continuous outcomes. ORs were estimated using weighted logistic regression with a logit link; RDs using weighted logistic regression with an identity link; and MDs using weighted linear regression. All models incorporated propensity score overlap weights

AVP: arginine vasopressin; NE: norepinephrine; CI confidence interval

To assess the robustness of our findings, we conducted a sensitivity analysis excluding patients receiving mechanical circulatory support (IABP, VV-ECMO, and VA-ECMO) at vasopressor cessation. Both the primary and secondary outcomes remained consistent with the main analysis (Table S1).

### Subgroup analysis

#### Septic shock

Among 267 patients with septic shock, 157 discontinued AVP first and 110 discontinued NE first. The baseline characteristics (Table S2) and clinical parameters at the time of the first agent cessation (Table S3) were balanced after weighting. In the weighted cohort, the incidence of hypotension was 16% in the AVP discontinued first group, and 25% in the NE discontinued first group (odds ratio 1.7, 95%CI 0.8, 4.0; risk difference 8.9%, 95%CI − 4.4, 22.3; *P* = 0.19). Secondary outcomes were consistent with those of the overall cohort (Table S4).

#### Cardiogenic shock

Among 88 patients with cardiogenic shock, 44 discontinued AVP first and 44 discontinued NE first. Due to the small sample size, we were unable to achieve adequate covariate balance using overlap weighting, and, therefore, report unadjusted results only. The baseline characteristics are shown in Table S5, and the clinical parameters at the time of the first agent cessation are shown in Table S6. In the unadjusted cohort, the incidence of hypotension was 32% in the AVP discontinued first group and 30% in the NE discontinued first group (Table S7).

### Exploratory analysis

**Fig. 2:**
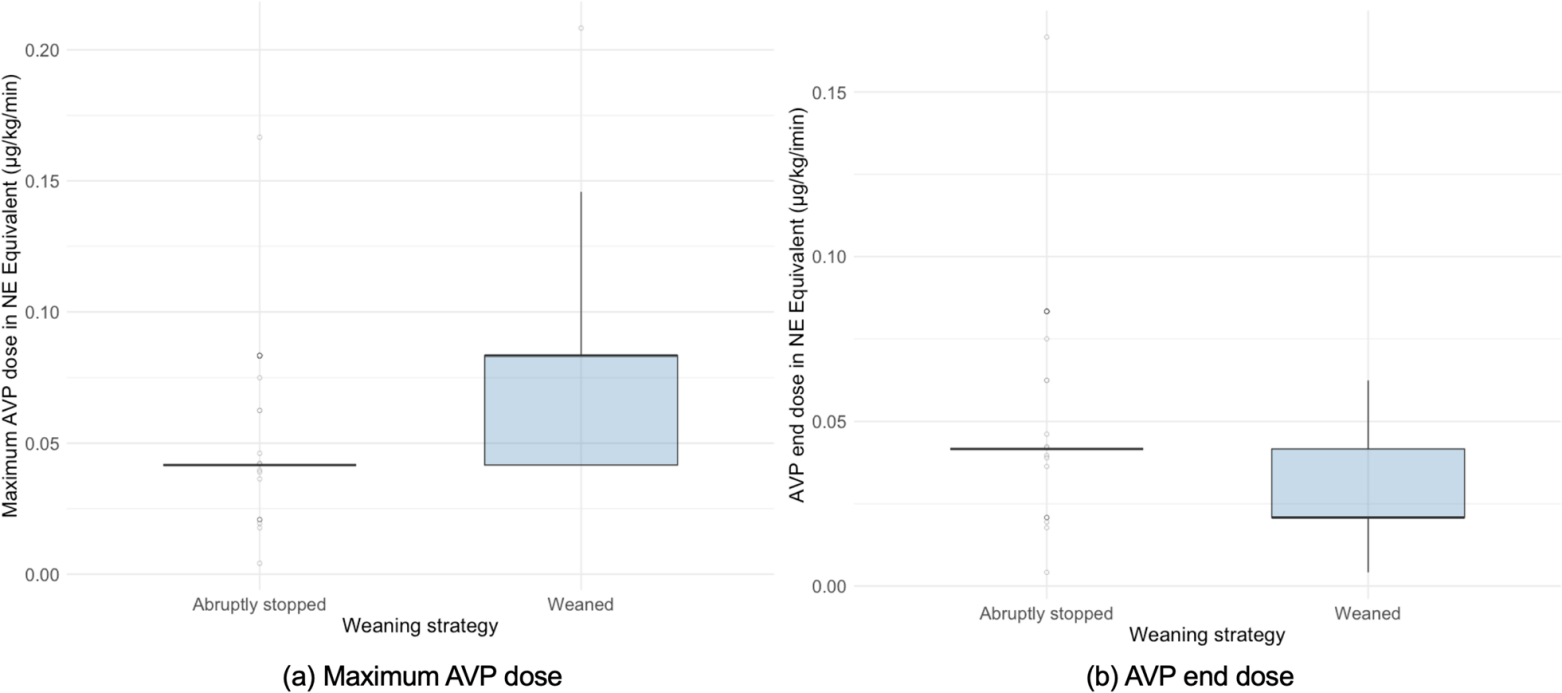
Maximum and end doses of arginine vasopressin (norepinephrine-equivalent) by weaning strategy in the arginine vasopressin discontinued first group. Box plots compare (**a**) the maximum arginine vasopressin (AVP) dose and (**b**) the AVP end dose, both converted to norepinephrine-equivalent units (μg/kg/min), between patients who underwent abrupt discontinuation vs gradual weaning of AVP in the AVP discontinued first group. AVP: arginine vasopressin; NE: norepinephrine

In the AVP discontinued first group, hypotension occurred in 27% of patients with abrupt discontinuation (*n* = 98) and 15% with gradual weaning (*n* = 195) in unadjusted analysis. Among patients in the AVP discontinued first group, the AVP end dose (NE equivalent) was 0.042 μg/kg/min in the abruptly discontinued group and 0.021 μg/kg/min (IQR 0.021–0.042) in the weaned group. The maximum AVP dose was lower in the abruptly discontinued group (0.042 μg/kg/min) than in the weaned group (median 0.083 μg/kg/min; IQR 0.042–0.083) (Fig. 2).

## Discussion

In this study, after balancing patient characteristics, including the vasopressor end dose immediately before discontinuation, with propensity score overlap weighting, the order of vasopressor discontinuation was not associated with a significant difference in the incidence of hypotension or other clinical outcomes. These findings were consistent in both the overall cohort and the septic shock subgroup.

### Comparison with previous studies

Previous observational studies have generally shown a higher incidence of hypotension when AVP was discontinued before NE in patients with septic shock. Bauer et al. reported a significantly higher incidence of hypotension within 24 h when AVP was discontinued first (55.6% vs 15.6%, *P* = 0.008) [6], and similar trends were observed by Mussallam [7], Hammond [8], Bissell [9] and Song [10]. Although Sacha et al. observed no significant difference overall (54.8% vs 49.8%, *P* = 0.28), early hypotension risk was higher with AVP discontinuation in a Cox model [14]. A recently published study by Starkel et al. evaluated rebound hypotension within 6 h of vasopressor discontinuation and found no significant difference; similar findings were observed at 12 and 24 h [15]. In this study, hypotension rates in the NE discontinued first group were consistent with prior reports [6–10], but notably lower in the AVP discontinued first group [6–10, 14, 15]. One possible explanation is that AVP tapering was more common in our cohort (67%), whereas tapering was infrequent in prior studies (14.2% in Sacha et al.) [14], 31.6% in Bissell et al. [9], 41.3% and 16.9% in Starkel et al. [15]. Furthermore, our analysis includes NE-equivalent vasopressor end dose as a covariate, and even after weighting, the incidence of hypotension remained comparable between the groups. These suggests that higher AVP end doses may have contributed to the findings of prior studies [6–10, 14, 15].

A randomized controlled trial by Mallmann [16] et al. supports this interpretation. In that study, AVP was tapered in 92% of patients, and there was no significant difference in the incidence of hypotension between the AVP discontinued first group and the NE discontinued first group (25.6% vs 43.6%, *P* = 0.153). A post hoc analysis showed a trend toward more hypotension with abrupt AVP discontinuation compared to weaning (66.7% vs 22.2%, *P* = 0.156), suggesting that discontinuation strategy may influence outcomes [16]. Similarly, our unadjusted data showed lower incidence of hypotension with AVP weaning. In our cohort, abrupt discontinuation occurred at lower AVP end doses (median 0.017 U/min; NE-equivalent 0.042 μg/kg/min) than in Mallmann’s trial (0.03 U/min) [16], possibly explaining the lower hypotension rate (27% vs 66.6% [16]).

Previous studies on AVP in vasodilatory shock often employed tapering protocols. The VASST trial tapered AVP by 0.3 U every hour after achieving the target mean arterial pressure [17], and Argenziano et al. tapered AVP to 0.01–0.02 U/min before discontinuation [18]. Tapering of AVP was also described in the study protocols of Dünser et al. [19] and the VANCS trial [20]. In contrast, a recent survey found that about 70% of clinicians discontinue AVP abruptly [21]. In a retrospective study comparing abrupt and tapered AVP discontinuation in patients with septic shock, the incidence of hypotension was similar (39.7% vs 41.7%) [13]; however, the end dose in the tapered group was higher than in this study (0.02) [13] vs 0.008 U/min), potentially explaining the higher incidence of hypotension despite tapering.

### Physiological and pharmacological interpretation

Our findings suggest that in patients recovering from shock who are receiving both AVP and NE, adequate tapering of AVP allows for a similar risk of hypotension as with NE-first discontinuation. This observation indicates that dose reduction when discontinuing AVP may be clinically relevant and could inform individualized weaning approaches. The pharmacologic profile of AVP may explain these clinical observations. In septic shock and vasodilatory shock following cardiac surgery, plasma AVP levels decline over time, resulting in relative deficiency [18, 22, 23]. Although AVP exerts minimal vasoconstrictive effects in normotensive individuals, it induces a strong pressor response in hypotensive states [22, 24], supporting its role as hormone replacement in septic shock [25]. However, its effectiveness even in the absence of AVP deficiency [26] and regardless of baseline AVP levels [27] suggests that AVP also acts as a titratable vasopressor.

Since AVP levels may remain low for several days in septic shock [17, 28], abrupt discontinuation during these periods could unmask a deficiency and cause hemodynamic instability, suggesting a need for tapering. From a pharmacokinetic perspective, vasopressin has a relatively short half-life (10 to 35 min) [29], so abrupt discontinuation may cause a rapid drop in plasma levels and trigger hypotension. A dose of 0.01 U/min can restore AVP to stress-induced physiological levels (30 pg/mL) and provide effective vasopressor support [22], making it a practical target for tapering.

### Strengths and limitations

This study’s strengths include the use of overlap weighting with propensity scores to balance baseline characteristics and reduce confounding, and the novel evaluation of vasopressor end dose in relation to hypotension. Furthermore, to our knowledge, no prior study has systematically examined the association between AVP tapering and the incidence of hypotension. Although the analysis was exploratory and unadjusted, it may provide a foundation for future research.

However, several Limitations should be noted. First, a post hoc power calculation showed that with 262 patients per group we had 80% power only to detect differences ≥ 11%, whereas the observed gap was just 3%, indicating this study may be underpowered and at risk for a type II error. Future studies with larger sample sizes will be necessary to reliably detect smaller differences between groups. Second, this was a retrospective single-center observational study, which may limit the generalizability. Third, while overlap weighting adjusts for measured covariates, unmeasured confounding may remain. Fourth, there was no standardized protocol for the initiation or discontinuation of vasopressors, and these decisions were made by the bedside clinicians, potentially introducing bias. Fifth, we could not identify patients transitioned to palliative care, which may have influenced outcomes. Finally, we selected the occurrence of hypotension as the primary outcome; however, transient hypotension that resolves promptly may not necessarily lead to clinically meaningful consequences. Nevertheless, the clinical characteristics and treatment practices in this study were consistent with prior studies [6–10, 14, 15], supporting its generalizability. The absence of a standardized protocol reflects real-world practice, enhancing the clinical relevance of our findings. In addition, selecting hypotension as the primary outcome is consistent with previous investigations of vasopressor discontinuation, reinforcing the validity and comparability of our study design.

## Conclusions

In this retrospective study of patients recovering from shock who received AVP and NE, we found no significant difference in the incidence of hypotension within 24 h of vasopressor cessation between the AVP discontinued first group and NE discontinued first group. These findings suggest that the discontinuation order of AVP and NE may not be a major determinant of hypotension risk.

## Supplementary Information

Additional file 1: Figure S1: Propensity score distributions before and after overlap weighting. Histograms illustrate the distribution of estimated propensity scores for the AVP discontinued first group and the NE discontinued first groupbefore andafter applying overlap weighting. AVP, Arginine vasopressin; NE, Norepinephrine

Additional file 2: Table S1. Summary of clinical outcomes in subgroup without mechanical circulatory support

Additional file 3: Table S2. Baseline characteristics at ICU admission in sepsis subgroup

Additional file 4: Table S3. Variables at the first vasopressor cessation in sepsis subgroup

Additional file 5: Table S4. Summary of clinical outcomes in septic shock subgroup

Additional file 6: Table S5. Baseline characteristics at ICU admission in cardiogenic shock subgroup

Additional file 7: Table S6. Variables at the first vasopressor cessation in cardiogenic shock subgroup

Additional file 8: Table S7. Summary of clinical outcomes in cardiogenic shock subgroup

## Data Availability

The data sets used and analyzed during the current study are available from the corresponding author on reasonable request.

## Abbreviations

ICU: Intensive care unit
NE: Norepinephrine
MAP: Mean arterial pressure
AVP: Arginine vasopressin
APACHE: Acute physiology and chronic health evaluation
SOFA: Sequential organ failure assessment
IABP: Intra-aortic balloon pumping
VV-ECMO: Veno-venous extracorporeal membrane oxygenation
VA-ECMO: Veno-arterial extracorporeal membrane oxygenation
RRT: Renal replacement therapy
HR: Heart rate
SMD: Standardized mean difference
