# Supplementary figures and images for: Strategies for discontinuing vasopressin and norepinephrine during the recovery phase of shock: a single-center retrospective study
Source: J Intensive Care. 2025 Sep 30;13:52. doi: 10.1186/s40560-025-00823-w (PMC12487481; doi:10.1186/s40560-025-00823-w)

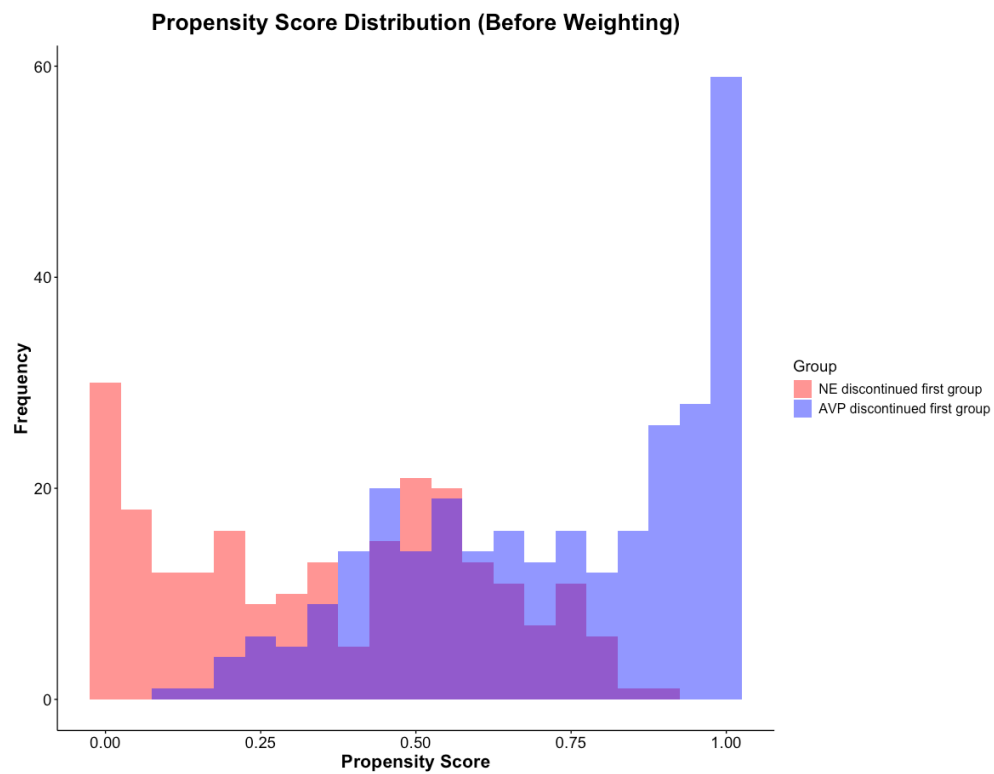

(a) Before weighting

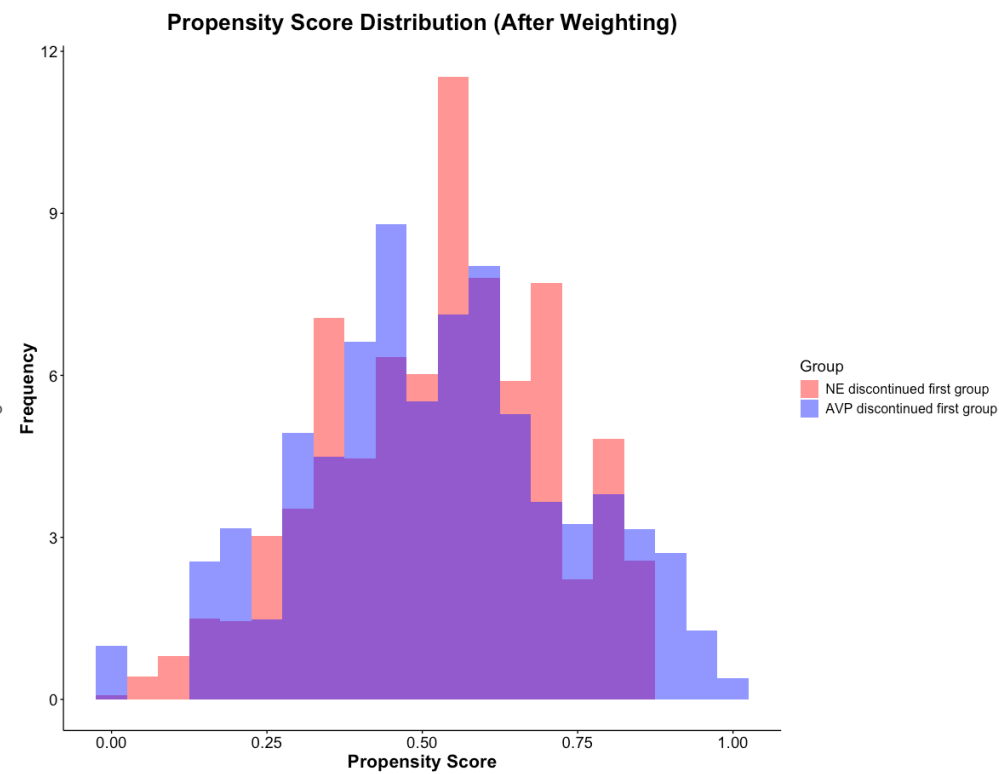

(b) After weighting

Supplement: Supplementary file 1 — Additional file 1: Figure S1: Propensity score distributions before and after overlap weighting. Histograms illustrate the distribution of estimated propensity scores for the AVP discontinued first group and the NE discontinued first groupbefore andafter applying overlap weighting. AVP, Arginine vasopressin; NE, Norepinephrine [file 40560_2025_823_MOESM1_ESM.pdf]
