# Supplementary material for: Strategies for discontinuing vasopressin and norepinephrine during the recovery phase of shock: a single-center retrospective study
Source: J Intensive Care. 2025 Sep 30;13:52. doi: 10.1186/s40560-025-00823-w (PMC12487481; doi:10.1186/s40560-025-00823-w)
Supplement: Supplementary file 3 — Additional file 3: Table S2. Baseline characteristics at ICU admission in sepsis subgroup [file 40560_2025_823_MOESM3_ESM.docx]

Table S2. Baseline characteristics at ICU admission in septic shock subgroup (Before and after overlap weighting)

|  | **Unadjusted cohort** | | | | **Weighted cohort** | | |
| --- | --- | --- | --- | --- | --- | --- | --- |
| **Variables** | **Overall**  **n = 267** | **AVP first**  **n = 157** | **NE first**  **n = 110** | **SMD** | **AVP first** | **NE first** | **SMD** |
| **Age (year)** | 72 (64–79) | 73 (65–79) | 71 (61–79) | 0.071 | 74 (64–79) | 73 (61–82) | <0.001 |
| **Admission Type** |  |  |  |  |  |  |  |
| Emergency Department | 86 (32) | 48 (31) | 38 (35) | 0.040 | 10.6 (32) | 10.6 (32) | <0.001 |
| Elective surgery | 4 (2) | 2 (1) | 2 (2) | 0.005 | 0.5 (1) | 0.5 (1) | <0.001 |
| Emergency surgery | 36 (14) | 21 (13) | 15 (14) | 0.003 | 4.5 (14) | 4.5 (14) | <0.001 |
| Transferred | 24 (9) | 14 (9) | 10 (9) | 0.002 | 1.7 (5) | 1.7 (5) | <0.001 |
| Ward | 117 (44) | 72 (46) | 45 (41) | 0.050 | 15.7 (48) | 15.7 (48) | <0.001 |
| **Male** | 178 (67) | 112 (71) | 66 (60) | 0.113 | 21.6 (65) | 21.6 (65) | <0.001 |
| **Weight (kg)** | 58.0 (50.0–66.4) | 58.0 (51.0–66.6) | 58.5 (48.9–65.8) | 0.096 | 57.9 (50.8–64.6) | 58.5 (50.0–64.8) | <0.001 |
| **Height (cm)** | 162 (155–170) | 162 (157–170) | 161 (154–170) | 0.140 | 161 (156–168) | 161 (155–170) | <0.001 |
| **Disease Category** |  |  |  |  |  |  |  |
| Cardiovascular | 0 (0) | 0 (0) | 0 (0) | <0.001 | 0 (0) | 0 (0) | <0.001 |
| Respiratory | 83 (31) | 48 (31) | 35 (32) | 0.012 | 8.0 (24) | 8.0 (24) | <0.001 |
| Gastrointestinal | 76 (29) | 42 (27) | 34 (31) | 0.042 | 11.3 (34) | 11.3 (34) | <0.001 |
| Neurological | 2 (1) | 2 (1) | 0 (0) | 0.013 | 0.0 (0) | 0.0 (0) | <0.001 |
| Others | 106 (40) | 65 (41) | 41 (37) | 0.041 | 13.8 (42) | 13.8 (42) | <0.001 |
| **Chronic Diseases** |  |  |  |  |  |  |  |
| Respiratory Failure | 4 (2) | 2 (1) | 2 (2) | 0.005 | 0.2 (1) | 0.2 (1) | <0.001 |
| Liver Cirrhosis | 7 (3) | 0 (0) | 7 (6) | 0.064 | 0.0 (0) | 0.0 (0) | <0.001 |
| Hematological Malignancy | 28 (11) | 17 (11) | 11 (10) | 0.008 | 3.0 (9) | 3.0 (9) | <0.001 |
| Cancer Metastasis | 17 (6) | 14 (9) | 3 (3) | 0.062 | 1.1 (3) | 1.1 (3) | <0.001 |
| Immunosuppression | 56 (21) | 33 (21) | 23 (21) | 0.001 | 6.8 (21) | 6.8 (21) | <0.001 |
| Maintenance Dialysis | 15 (6) | 11 (7) | 4 (4) | 0.034 | 1.2 (4) | 1.2 (4) | <0.001 |
| **APACHE Ⅲ score** | 106 (91–124) | 106 (91–121) | 106 (91–126) | 0.035 | 106 (91–124) | 105 (91–126) | <0.001 |
| **Creatinine (mg/dL)** | 0.9 (0.9–1.9) | 0.9 (0.9–1.9) | 0.9 (0.9–1.7) | 0.016 | 0.9 (0.9–1.9) | 0.9 (0.9–1.7) | <0.001 |
| **Lactate (mmol/L)** | 2.0 (1.3–3.6) | 2.0 (1.3–3.3) | 1.6 (1.3–4.1) | 0.029 | 2.1 (1.3–4.2) | 2.1 (1.3–4.1) | <0.001 |
| **PaO_2_/F_I_O_2_** | 265 (185–265) | 265 (174–265) | 265 (216–265) | 0.086 | 265 (186–265) | 265 (190–276) | <0.001 |
| **Cortisol use** | 230 (86) | 140 (89) | 90 (82) | 0.074 | 28.9 (87) | 28.9 (87) | <0.001 |

Categorical variables are presented as n (%), and continuous variables are presented as median (IQR).

APACHE, Acute Physiology and Chronic Health Evaluation; AVP, Arginine vasopressin; NE, Norepinephrine; SMD, Standardized Mean Difference.
