# Supplementary material for: Strategies for discontinuing vasopressin and norepinephrine during the recovery phase of shock: a single-center retrospective study
Source: J Intensive Care. 2025 Sep 30;13:52. doi: 10.1186/s40560-025-00823-w (PMC12487481; doi:10.1186/s40560-025-00823-w)
Supplement: Supplementary file 4 — Additional file 4: Table S3. Variables at the first vasopressor cessation in sepsis subgroup [file 40560_2025_823_MOESM4_ESM.docx]

Table S3. Variables at the first vasopressor cessation in septic shock subgroup (Before and after overlap weighting)

|  | **Unadjusted cohort** | | | | **Weighted cohort** | | |
| --- | --- | --- | --- | --- | --- | --- | --- |
| **Variables** | **Overall**  **n = 267** | **AVP first**  **n = 157** | **NE first**  **n = 110** | **SMD** | **AVP first** | **NE first** | **SMD** |
| **Lactate (mmol/L)** | 2.1 (1.5–3.3) | 1.9 (1.5–3.0) | 2.3 (1.7–4.2) | 0.340 | 2.0 (1.5–3.0) | 2.0 (1.6–3.0) | <0.001 |
| **SOFA Total**  **without cardiovascular** | 8 (6–10) | 8 (6–11) | 8 (6–10) | 0.050 | 8 (6–10) | 8 (6–9) | <0.001 |
| **SOFA Respiratory system** | 2 (1–3) | 2 (1–3) | 2 (1–2) | 0.050 | 2 (1–2) | 2 (1–2) | 0.050 |
| **SOFA Coagulation** | 2 (0–3) | 2 (0–3) | 2 (0–2) | 0.111 | 2 (0–3) | 2 (1–3) | 0.072 |
| **SOFA Liver** | 0 (0–1) | 0 (0–1) | 0 (0–1) | 0.023 | 0 (0–1) | 0 (0–1) | 0.012 |
| **SOFA Central nervous system** | 2 (2–3) | 2 (2–3) | 2 (2–3) | 0.044 | 2 (2–3) | 2 (1–3) | 0.006 |
| **SOFA Renal function** | 2 (1–4) | 2 (1–4) | 2 (1–4) | 0.023 | 2 (1–3) | 1 (1–3) | 0.033 |
| **IABP used** | 0 (0) | 0 (0) | 0 (0) | <0.001 | 0 (0.0) | 0 (0.0) | <0.001 |
| **VV-ECMO used** | 8 (3) | 3 (2) | 5 (5) | 0.026 | 0.3 (1) | 0.3 (1) | <0.001 |
| **VA-ECMO used** | 5 (2) | 4 (3) | 1 (1) | 0.016 | 0.2 (1) | 0.2 (1) | <0.001 |
| **RRT used** | 64 (24) | 43 (27) | 21 (19) | 0.083 | 6.1 (19) | 6.1 (19) |  |
| **Mean Arterial Pressure**  **(mmHg)** | 74 (68–80) | 72 (68–79) | 77 (68–80) | 0.210 | 73 (69–79) | 75 (70–80) | <0.001 |
| **Heart Rate (bpm)** | 88 (75–103) | 87 (75–100) | 90 (76–106) | 0.175 | 86 (75–99) | 88 (70–101) | <0.001 |
| **Cumulative fluid balance**  **before cessation (mL)** | 3,401 (1,557–5,753) | 3,558 (1,713–6,112) | 2,794 (1,387–5,444) | 0.300 | 2,916 (1,613–5,310) | 3,084 (1,618–5,210) | <0.001 |
| **Vasopressor end dose**  **in NEE (μg/kg/min)** | 0.04 (0.02–0.04) | 0.04 (0.02–0.04) | 0.04 (0.02–0.10) | 3.366 | 0.04 (0.02–0.04) | 0.03 (0.02–0.04) | <0.001 |
| **The other vasopressor dose**  **at cessation in NEE**  **(μg/kg/min)** | 0.06 (0.04–0.10) | 0.08 (0.04–0.14) | 0.04 (0.04–0.08) | 2.917 | 0.04 (0.02–0.07) | 0.04 (0.04–0.08) | <0.001 |
| **Duration from ICU admission to first vasopressor cessation**  **(hours)** | 26 (14­–50) | 27 (16–50) | 24 (10–49) | 0.466 | 27 (15–42) | 33 (17–51) | <0.001 |

Categorical variables are presented as n (%), and continuous variables are presented as median (IQR).

SOFA, Sequential Organ Failure Assessment; IABP, Intra-Aortic Balloon Pumping; VV-ECMO, Veno-Venous Extracorporeal Membrane Oxygenation; VA-ECMO, Veno-Arterial Extracorporeal Membrane Oxygenation; RRT, Renal Replacement Therapy; NEE, Norepinephrine equivalent: AVP (U/min) × 2.5 = NE (μg/kg/min); ICU, Intensive care unit; AVP, Arginine vasopressin; NE, Norepinephrine; SMD, Standardized Mean Difference.
