# Supplementary material for: Strategies for discontinuing vasopressin and norepinephrine during the recovery phase of shock: a single-center retrospective study
Source: J Intensive Care. 2025 Sep 30;13:52. doi: 10.1186/s40560-025-00823-w (PMC12487481; doi:10.1186/s40560-025-00823-w)
Supplement: Supplementary file 5 — Additional file 5: Table S4. Summary of clinical outcomes in septic shock subgroup [file 40560_2025_823_MOESM5_ESM.docx]

Table S4. Summary of clinical outcomes in septic shock subgroup

|  | **Unadjusted cohort** | | **weighted cohort** | | | |
| --- | --- | --- | --- | --- | --- | --- |
|  | **AVP first** | **NE first** | **AVP first** | **NE first** | **Effect estimates (95%CI)** | ***P*** |
| **Primary outcome** |  |  |  |  |  |  |
| Hypotension | 18 | 26 | 16 | 25 | OR 1.7 (0.8, 4.0); RD 8.9 (-4.4, 22.3) | 0.19 |
| **Secondary outcome** |  |  |  |  |  |  |
| Hospital mortality | 41 | 40 | 32 | 34 | OR 1.1 (0.5, 2.2); RD 1.6 (-14.5, 17.8) | 0.84 |
| ICU mortality | 27 | 31 | 17 | 23 | OR 1.5 (0.6, 3.5); RD 6.0 (-7.7, 19.6) | 0.39 |
| Hospital length of stay (days) | 59 | 41 | 61 | 49 | MD -11.7 (-38.5, 15.1) | 0.39 |
| ICU length of stay (days) | 16 | 10 | 14 | 9 | MD -4.9 (-9.9, 0.1) | 0.06 |
| Incidence of new onset  atrial fibrillation | 15 | 13 | 13 | 14 | OR 1.0 (0.4, 2.5); RD 0.2 (-0.1, 0.1) | 0.97 |
| Cumulative fluid balance  after cessation (mL) | 5,636 | 2,780 | 3,604 | 1,595 | MD -2009.0 (-5083.6, 1065.6) | 0.20 |
| Total vasoactive medication duration (days) | 8 | 5 | 6 | 4 | MD -1.8 (-5.5, 2.0) | 0.35 |

Categorical variables are presented as percentages, and continuous variables as means.

Effect estimates are presented as odds ratios (ORs) and risk differences (RDs) for binary outcomes, and as mean differences (MDs) for continuous outcomes. ORs were estimated using weighted logistic regression with a logit link; RDs using weighted logistic regression with an identity link; and MDs using weighted linear regression. All models incorporated propensity score overlap weights.

AVP, Arginine vasopressin; NE, Norepinephrine; CI, confidence interval.
