# Supplementary material for: Strategies for discontinuing vasopressin and norepinephrine during the recovery phase of shock: a single-center retrospective study
Source: J Intensive Care. 2025 Sep 30;13:52. doi: 10.1186/s40560-025-00823-w (PMC12487481; doi:10.1186/s40560-025-00823-w)
Supplement: Supplementary file 6 — Additional file 6: Table S5. Baseline characteristics at ICU admission in cardiogenic shock subgroup [file 40560_2025_823_MOESM6_ESM.docx]

Table S5. Baseline characteristics at ICU admission in cardiogenic shock subgroup (Unadjusted cohort)

| **Variables** | **Overall**  **n = 88** | **AVP first**  **n = 44** | **NE first**  **n = 44** | **SMD** |
| --- | --- | --- | --- | --- |
| **Age (year)** | 73 (63–79) | 75 (64–79) | 70 (63–79) | 0.026 |
| **Admission Type** |  |  |  | 0.376 |
| Emergency Department | 28 (32) | 15 (34) | 13 (30) |  |
| Elective surgery | 19 (22) | 12 (27) | 7 (16) |  |
| Emergency surgery | 13 (15) | 6 (14) | 7 (16) |  |
| Transferred | 9 (10) | 3 (7) | 6 (14) |  |
| Ward | 19 (22) | 8 (18) | 11 (25) |  |
| **Male** | 64 (73) | 31 (71) | 33 (75) | 0.102 |
| **Weight (kg)** | 60.0 (50.0–70.5) | 58.9 (50.0–70.0) | 61.9 (50.0–75.5) | 0.188 |
| **Height (cm)** | 160 (155–168) | 160 (155–169) | 163 (157–168) | 0.105 |
| **Chronic Diseases** |  |  |  |  |
| Respiratory Failure | 2 (2) | 1 (2) | 1 (2) | <0.001 |
| Liver Cirrhosis | 1 (1) | 0 (0) | 1 (2) | 0.216 |
| Hematological Malignancy | 0 (0) | 0 (0) | 0 (0) | <0.001 |
| Cancer Metastasis | 1 (1) | 1 (2) | 0 (0) | 0.216 |
| Immunosuppression | 3 (3) | 1 (2) | 2 (5) | 0.125 |
| Maintenance Dialysis | 10 (11) | 3 (7) | 7 (16) | 0.289 |
| **APACHE Ⅲ score** | 106 (78–140) | 108 (77–140) | 102 (80–137) | 0.076 |
| **Creatinine (mg/dL)** | 0.9 (0.9–1.6) | 0.9 (0.9–1.6) | 1.0 (0.8–1.6) | 0.175 |
| **Lactate (mmol/L)** | 2.5 (1.3–5.9) | 2.5 (1.3–5.9) | 2.5 (1.3–6.0) | 0.070 |
| **PaO_2_/F_I_O_2_** | 265 (188–275) | 265 (173–265) | 265 (221–338) | 0.294 |
| **Cortisol use** | 53 (60) | 26 (59) | 27 (61) | 0.046 |

Categorical variables are presented as n (%), and continuous variables are presented as median (IQR).

APACHE, Acute Physiology and Chronic Health Evaluation; AVP, Arginine vasopressin; NE, Norepinephrine; SMD, Standardized Mean Difference.
