# Supplementary material for: Strategies for discontinuing vasopressin and norepinephrine during the recovery phase of shock: a single-center retrospective study
Source: J Intensive Care. 2025 Sep 30;13:52. doi: 10.1186/s40560-025-00823-w (PMC12487481; doi:10.1186/s40560-025-00823-w)
Supplement: Supplementary file 7 — Additional file 7: Table S6. Variables at the first vasopressor cessation in cardiogenic shock subgroup [file 40560_2025_823_MOESM7_ESM.docx]

Table S6. Variables at the first vasopressor cessation in cardiogenic shock subgroup (Unadjusted cohort)

| **Variables** | **Overall**  **n = 88** | **AVP first**  **n = 44** | **NE first**  **n = 44** | **SMD** |
| --- | --- | --- | --- | --- |
| **Lactate (mmol/L)** | 2.0 (1.4–3.8) | 2.5 (1.5–4.1) | 1.8 (1.4–3.1) | 0.125 |
| **SOFA Total**  **without cardiovascular** | 9 (7–11) | 9 (7–11) | 9 (6–11) | 0.118 |
| **SOFA Respiratory system** | 2 (1–3) | 2 (1–3) | 2 (1–2) | 0.090 |
| **SOFA Coagulation** | 1 (0–2) | 2 (1–2) | 1 (0–2) | 0.064 |
| **SOFA Liver** | 0 (0–2) | 0 (0–2) | 0 (0–1) | 0.085 |
| **SOFA Central nervous system** | 3 (2–4) | 3 (2–4) | 3 (2–4) | 0.165 |
| **SOFA Renal function** | 3 (1–4) | 2 (1–4) | 3 (0–4) | 0.027 |
| **IABP used** | 6 (7) | 4 (9) | 2 (5) | 0.181 |
| **VV-ECMO used** | 0 (0) | 0 (0) | 0 (0) | <0.001 |
| **VA-ECMO used** | 8 (9) | 6 (14) | 2 (5) | 0.320 |
| **RRT used** | 23 (26) | 10 (23) | 13 (30) | 0.156 |
| **Mean Arterial Pressure**  **(mmHg)** | 72 (68–79) | 72 (68–76) | 74 (69–82) | 0.159 |
| **Heart Rate (bpm)** | 86 (73–98) | 91 (78–101) | 80 (68–95) | 0.422 |
| **Cumulative fluid balance**  **before cessation (mL)** | 2,992 (1,339–6,979) | 3,460 (1,715–7,270) | 2,627 (1,092–5,655) | 0.008 |
| **Vasopressor end dose**  **in NEE (μg/kg/min)** | 0.03 (0.02–0.04) | 0.02 (0.02–0.04) | 0.03 (0.02–0.07) | 0.596 |
| **The other vasopressor dose**  **at cessation in NEE**  **(μg/kg/min)** | 0.07 (0.04–0.10) | 0.10 (0.06–0.20) | 0.04 (0.04–0.08) | 0.797 |
| **Duration from ICU admission to**  **first vasopressor cessation**  **(hours)** | 42 (20­–65) | 42 (23–64) | 42 (15–68) | 0.416 |

Categorical variables are presented as n (%), and continuous variables are presented as median (IQR).

SOFA, Sequential Organ Failure Assessment; IABP, Intra-Aortic Balloon Pumping; VV-ECMO, Veno-Venous Extracorporeal Membrane Oxygenation; VA-ECMO, Veno-Arterial Extracorporeal Membrane Oxygenation; RRT, Renal Replacement Therapy; NEE, Norepinephrine equivalent: AVP (U/min) × 2.5 = NE (μg/kg/min); ICU, Intensive care unit; AVP, Arginine vasopressin; NE, Norepinephrine; SMD, Standardized Mean Difference.
