# Supplementary material for: Strategies for discontinuing vasopressin and norepinephrine during the recovery phase of shock: a single-center retrospective study
Source: J Intensive Care. 2025 Sep 30;13:52. doi: 10.1186/s40560-025-00823-w (PMC12487481; doi:10.1186/s40560-025-00823-w)
Supplement: Supplementary file 8 — Additional file 8: Table S7. Summary of clinical outcomes in cardiogenic shock subgroup [file 40560_2025_823_MOESM8_ESM.docx]

Table S7. Summary of clinical outcomes in cardiogenic shock subgroup (Unadjusted cohort)

|  | **AVP first** | **NE first** |
| --- | --- | --- |
| **Primary outcome** |  |  |
| Hypotension | 32 | 30 |
| **Secondary outcome** |  |  |
| Hospital mortality | 41 | 66 |
| ICU mortality | 34 | 30 |
| Hospital length of stay (days) | 45 | 45 |
| ICU length of stay (days) | 17 | 20 |
| Incidence of new onset  atrial fibrillation | 14 | 11 |
| Cumulative fluid balance  after cessation(mL) | 3,323 | 3,941 |
| Total vasoactive medication duration (days) | 9 | 9 |

Categorical variables are presented as %, and continuous variables are presented as mean.

AVP, Arginine vasopressin; NE, Norepinephrine.
